# A qualitative study exploring contributors to the success of a community of practice in rehabilitation

**DOI:** 10.1186/s12909-021-02711-x

**Published:** 2021-05-17

**Authors:** Jennifer L. Moore, Cato Bjørkli, Richard Tidemann Havdahl, Linn Lien Lømo, Mari Midthaug, Marita Skjuve, Mari Klokkerud, Jan E. Nordvik

**Affiliations:** 1 Regional Center for Knowledge translation in Rehabilitation South Eastern Region, Sunnaas Sykehus, Nesodden, Oslo, Norway; 2 Institute for Knowledge Translation, Carmel, IN USA; 3 University of Oslo, Department of Psychology, Oslo, Norway; 4 AFF at the Norwegian School of Economics, Oslo, Norway; 5 Hartmark Executive Search, Oslo, Norway; 6 Catosenteret Rehabilitation Center, Son, Norway

**Keywords:** Professional education, Community of Practice, Evidence-based practice, Translational medical research, Rehabilitation

## Abstract

**Background:**

Communities of Practice (CoPs) focus on learning, knowledge sharing, and creation, and research indicates they can improve healthcare performance. This article describes the development of a CoP that focused on synthesizing and adapting evidence in Physical Medicine and Rehabilitation (PM&R). This study aimed to investigate the CoP members’ experiences and perceived barriers and enablers of CoP success in the early phase of a CoP.

**Methods:**

Physical therapists and a physician (*n* = 10) volunteered for a CoP that synthesized literature of PM&R evidence. CoP members participated in education and training on critical appraisal and knowledge synthesis, practiced critical appraisal skills, and summarized literature. Three months after CoP initiation, semi-structured interviews were conducted to understand the CoP members’ experiences and reflections. Members also completed an online survey that included the Evidence-Based Practice Confidence scale (EPIC), questions related to CoP activities, and demographics before CoP initiation. We utilized the Capability, Opportunity, and Motivation Model of Behaviour (COM-B) to explore how these experiences related to the behavioral adaptation and participation.

**Results:**

Ten themes related to the potential contributors to CoP success and failure were identified. These included project management, technological solutions, efficacy, organizational support, interaction, the bigger picture, self-development, time, and motivation.

**Conclusions:**

Contributors to CoP success may include clearly articulated project goals and participant expectations, education and training, reliable technology solutions, organizational support, face-to-face communication, and good project management. Importantly, CoP members need time to participate in activities.

**Supplementary Information:**

The online version contains supplementary material available at 10.1186/s12909-021-02711-x.

## Implications for rehabilitation

- Communities of practice focus on learning, knowledge sharing, and creation, and could support the implementation of evidence-based practices in rehabilitation.
- Organizational support and dedicated time for CoP members may ensure active participation and maximize benefits.
- Communities of practice can be formed online with proper technology and project management.

## Background

Health services across the world spend billions of dollars annually on the development of evidence-based health interventions, but only a small fraction of these are implemented into practice [1]. The complex issue of translating research into healthcare practice is addressed within implementation science and knowledge translation. Research suggests that traditional approaches to implementation have been minimally successful, as systematic reviews indicate that time lags for translation of research into practice are greater than 17 years [2]. Studies have also found that patients receive only 55% of recommended care and approximately 30% of patients receive care that is not needed or potentially harmful [3, 4].

The healthcare sector has looked to other industries for new approaches to improve the delivery of high-quality health care. One strategy that has gained recognition in the business sector is the promotion and development of communities of practice (CoP). The term CoP was originally proposed by Lave and Wenger in 1991 [5], and was later described as a group of people who engage in collective learning as they interact regularly about a shared topic of interest [6]. CoPs resemble other collaborative networks, similar to clinical [7] and social-professional networks [8], and these terms are often used interchangeably [9]. However, as a distinct characteristic of CoPs, the groups focus on learning, knowledge sharing, and creation [8]. CoPs may vary in size, interaction forms, knowledge domain and whether they are self-organized (informal), or mandated and governed (formal).

Research in other sectors has revealed critical issues to consider while establishing collaborative networks. Schuck [10] successfully recruited interested stakeholders to a network for teachers, but a large proportion failed to participate after it was initiated. Nicolini and colleagues [11] suggest that a CoP might become a barrier to learning if not properly integrated into the organizational context. This suggests that the success of a CoP may be dependent on understanding factors influencing active participation and organizational integration. A CoP should be considered in a developmental perspective that evolves from an early stage (termed by Wenger [12] as the *potential* and *coalescing* phase) with few expectations or matured professional relationships between the members, to a habituated group of members with refined expectations and experiences with their common activity (the *matured* phase) [12].

A recent increase in research on CoPs in healthcare demonstrates the growing interest and perceived benefit of these groups [13, 14]. A systematic review by Ranmuthugala and colleagues [13], indicates that CoPs can improve healthcare performance by helping to implement evidence-based practices and achieving sustainable service improvements. While this research is promising, understanding the impact of a CoP in a specific field of medicine may be valuable since context, clinician training, and barriers vary by field and profession. In the field of physical medicine and rehabilitation (PM&R), early-stage CoPs were recently assessed for feasibility, barriers, and facilitators. A variety of formats have been used for meetings, including primarily in-person meetings [15, 16], primarily online meetings [17, 18], and a combination of in-person and online meetings [19, 20]. The focus of the CoPs has included the development of guidelines [15], development of general evidence-based practice recommendations [19], peer collaboration, mentoring and professional networking [17, 18], and conducting evidence synthesis and adaptation [20]. The reported barriers to CoP success included lack of knowledge related to critical appraisal [19], lack of organizational support [15, 19], problems related to technology [17], issues or preferences related to group interaction and communication [16, 17], and lack of group commitment [16]. Enablers included perceived benefits of the CoP, such as networking and mentoring opportunities, professional development, and group structure [16–19].

While this research provides information about format and focus of CoPs in PM&R, we are unaware of research on factors to consider during CoP development that may increase their success. Consideration of these factors during CoP development may increase the efficiency and effectiveness of the group. The objectives of the study were to investigate new CoP members’ experiences, perceived barriers, and enablers of CoP success during the early phases of a CoP. We also utilized the Capability, Opportunity, and Motivation Model of Behaviour (COM-B) framework to explore how these experiences related to the behavioral adaptation and participation in the early phase of a CoP.

## Methods

In this project, we studied experiences of participants in a newly developed CoP that aimed to support implementation of evidence-based practices in PM&R. The CoP, established by the Regional Center of Knowledge Translation in Rehabilitation in Olso, Norway, conducted critical appraisals of research articles, synthesized evidence, and developed recommendations for use of the evidence in clinical practice. The CoP members were required to collectively learn critical appraisal skills and methods to synthesize knowledge. Practitioners from different rehabilitation institutions in southeastern Norway were recruited and trained to participate in the project.

### CoP development and training

Participants, including physical therapists (*n* = 9) and a physician (*n* = 1), were known as the Knowledge Experts, performed CoP tasks in addition to regular work tasks and did not receive compensation for participating. A primary CoP goal was to produce one research summary within the first year of the project. In order to do so, the Knowledge Experts participated in education and training on how to collect, critically evaluate and summarize research evidence. In the second phase of the CoP, the Knowledge Experts decided on topics for the summaries and practiced the critical appraisal skills acquired, before reviewing the literature and producing the research summary in the final phase. The training consisted of e-learning courses and bi-monthly online videoconferences. An expert in knowledge translation from the United States led the project, in collaboration with a Norwegian project manager, and was responsible for the administration, coordination, and training of the CoP. The online conferences were conducted in English, the second language of the CoP members, due to the instructor’s primary language. Due to the members’ geographical location, interaction occurred primarily through a web-based project site and the bi-monthly video conferences. A two-day in-person meeting occurred 3 months after project initiation. Additional in-person training on knowledge translation was provided over a 3-day period approximately 1-year after the initiation of the project.

### Research design

After the Knowledge Experts were recruited, but before they participated in the education, training, or CoP meetings, they completed an online survey, including the evidence-based practice Confidence scale (EPIC) and demographic questions. The EPIC includes 11 questions that ask the participants to rate their confidence to perform various components of evidence-based practice, such as conducting literature searches, critical appraisals, and integration of research into practice and patient preferences [21]. Participants rate themselves on an 11-point scale with a range of 0 to 100%, and the total score reflects the mean of each of the individual items [21]. A low score indicates minimal confidence and 100% indicates complete confidence. The EPIC was developed and revised using feedback from health care providers, which established face and content validity [21]. In a sample of physical therapists, the EPIC also demonstrated excellent test-retest reliability (ICC = .89), excellent internal consistency (Cronbach’s alpha = .89), and acceptable construct validity [22]. Five additional questions were developed using EPIC’s structure to target areas covered in the Knowledge Expert education and training. The developed questions are referred to as the Knowledge Expert Questions and are listed in Table 1. This survey was administered to provide an estimate of the knowledge experts’ baseline confidence and knowledge related to evidence-based practice and the aims of the CoP.

**Table 1:** Survey questions and results

*EPIC. On a scale of 0% to 100%, how confident are you in your ability to:* (abbreviated questions are listed below)	Mean (SD) (*n*=9)
EPIC Total Score	57.9 (21.7)
Identify a gap in your knowledge … ?	82.2 (8.3)
Formulate a question to guide a literature search … ?	61.1 (15.3)
Effectively conduct an online literature … ?	62.2 (20.4)
Critically appraise the strengths and weaknesses of study methods … ?	50.0 (21.2)
Critically appraise the measurement properties standardized tests … ?	55.6 (22.4)
Interpret results such as t-tests or chi-square tests?	20.0 (19.4)
Interpret results such as linear or logistic regression?	20.0 (21.8)
Determine if evidence applies to your patient’s or client’s situation?	57.8 (21.1)
Ask your patient/client about needs, values and treatment preferences?	85.6 (8.8)
Decide on an appropriate course of action based on integrating the research, clinical judgment and patient or client preferences?	75.6 (18.1)
Continually evaluate the effect of your treatment on patient/client outcomes?	66.7 (25.5)
Knowledge Expert Questions	
KE questions, total score	39.1 (18.0)
Use the standard error of measurement to support interpretation of measurement results published in articles or from my own clinical practice?	26.7 (25.5)
Use the minimum detectable change to track a patient’s change over time?	28.9 (25.5)
Use the minimal clinical important difference to track a patient’s change over time?	23.3 (22.4)
Determine an appropriate dose of an intervention to provide to a patient?	63.3 (20)
Select an appropriate outcome measurement to assess change that results from an intervention?	53.3 (21.8)

To investigate the Knowledge Experts’ experiences, we conducted a qualitative study using semi-structured interviews to gain an in-depth understanding of the respondents’ CoP experiences and reflections [23]. The interviews were conducted 3 months after CoP initiation to ensure sufficient experience of the current early phase, while still avoiding retrospective reporting. An interview guide was developed and tested through pilot interviews. The questions were open-ended and based on the SWOT-framework, an organizing framework developed to facilitate reflection upon Strengths, Weaknesses, Opportunities and Threats (Supplementary file 1) [24]. This framework is explorative in nature, and participants were asked to reflect without restrictions along two dimensions of the project: positive-negative and past-future [25, 26]. We conducted individual in-depth semi-structured interviews at the participants’ workplace and during the two-day in-person meeting. Four researchers who completed extensive training on qualitative research conducted the interviews. Responses were re-stated and summarized back to the participant to ensure an accurate description of the participant’s experience [27].

### Recruitment

A convenience sample that consisted of recruitment of clinicians from the recently established Knowledge Expert CoP. We provided the Knowledge Experts with information about the project, including an overview of the survey and semi-structured interviews. This information was provided using two separate emails, sent 3 months apart, with an invitation to participate. The first email included a link to an online survey that provided an informed consent on the landing page. A later email described the purpose of the semi-structured interviews and an informed consent form. Clinicians also received the interview guide to familiarize and reflect upon the interview questions [28, 29].

### Data analysis

**Fig. 1:**
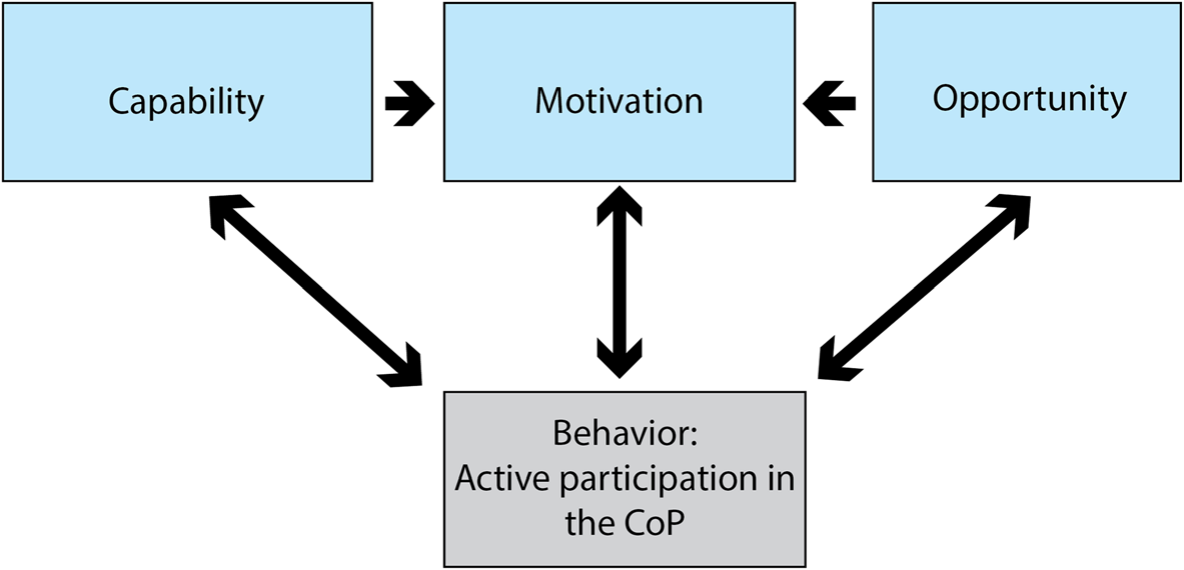
The Capability, Opportunity, and Motivation Model of Behavior

The semi-structured interviews were audio-recorded and later transcribed verbatim for analysis and de-identification. Four researchers analyzed themes with an inductive approach and then conducted a deductive analysis to classify themes using the COM-B model. This is a framework for behavioral change intervention that (Fig. 1) focuses on capability (C), opportunity (O) and motivation (M) to engage in the target behavior (B) [30]. *Capability* is defined as “the individual’s physical and psychological capacity to engage in the behavior.” [30] *Psychological capability* refers to knowledge or psychological skills that are necessary in order to engage in mental processes, whereas *physical capability* involves physical skill or strength [31]. *Opportunity* involves all factors outside the individual that can promote or prompt behavior. This can be either a *physical opportunity* given by the environment, for instance, time and resources or *social opportunities* afforded by interpersonal influences, social cues or cultural norms that influence the way we think. The last component, motivation is defined as “all brain processes that energize and direct behavior.” *Motivation* includes two subcategories: reflective and automatic. *Reflective motivation* denotes processes involving planning and evaluations, while *automatic motivation* refers to impulses and emotions which are not necessarily consciously recognized but can direct behavior [31]. The COM-B model represents that a specific behavior will occur only when a person has the capability and opportunity to engage in it. Further, the person is more motivated to enact that behavior than any other behaviors. Michie and colleagues [31] contend that the model can be utilized to understand or change the behavior of the individual, as well as the group and organizations. As illustrated in Fig. 1, motivation can indirectly influence capability and opportunity through behavior, while capability and opportunity influences motivation directly, as well as indirectly through behavior. Additionally, feedback from engaging in behaviors can influence all three components [30].

#### Inductive analysis

Inductive analysis was conducted using Braun and Clark’s approach to thematic analysis [32]. The researchers familiarized themselves with the transcripts, before reviewing these further and assigning initial descriptive codes to meaningful units of text. Two researchers coded each transcript and initial codes were compared. Discrepancies in the coding were resolved by reaching consensus through discussion [33]. After coding the entire dataset, codes were compared and categorized, and then combined into overarching themes. Themes and categories were reviewed and refined to ensure that they sufficiently captured nuances in the dataset. The final themes were renamed, and each theme was described based on inherent categories. All categories and themes were reviewed and discussed by the four researchers. To ensure the accuracy and validity of the themes, participants reviewed and confirmed the results and stated they did not require modifications.

#### Deductive analysis

Four researchers performed the deductive analysis by organizing themes into the COM-B model of behavior [30]. Each researcher placed themes into the model, and then compared results. When discrepancies occurred, consensus was reached through discussion.

#### Ethics

Ethical approval was obtained by the Data Protection Official at the University Hospital of Oslo. Participants reviewed and signed an informed consent document. Participants were able to withdraw from the study at any time, and confidentiality and anonymity were ensured by following the Data Protection Official’s guidelines for data collection, storage, and processing.

## Results

Nine of the ten Knowledge Experts responded to the survey, and the survey demographics and results are described in Tables 1 and 2. The average score on the initial EPIC was 57.9% (SD = 21.7), with the highest scoring item relating to asking patients/clients about needs, values and treatment preferences (85.6%, SD = 8.8). The lowest scoring items inquired about the interpretation of t-tests or chi-square tests (20%, SD = 19.4) and linear or logistic regressions (20%, SD = 21.8). For the Knowledge Expert questions, the average score was 39.1% (SD = 18.0), with the lowest scoring items relating to the use of research data, such as standard error of measurement, minimum detectable change, and minimum clinically important differences, to interpret measurement results in clinical practice.

**Table 2:** Participant demographics provided on the survey

Variable	Count
Number of participants	9
Age, count	
20–29 years	1
30–39 years	4
40–49 years	2
50+ years	2
Years in practice, mean (range)	
< 5 years	1
5–10 years 4	4
11–15 years 1	1
> 15 years 3	3
Highest Degree	
Bachelor	5
Master	1
Doctorate	0
Other	3
Seeking higher degree (in the future)	
Yes	5
No	1
Do not know	3
Regularly attend continuing education	
Yes	7
No	2
Taken eLearning course in last year	
Yes	3
No	6
Patient group most frequently treated	
Orthopedic	1
Neurologic	2
Cardiovascular /pulmonary	1
Musculoskeletal / pain	5

Ten Knowledge Experts provided consent (*n* = 10) to participate in the semi-structured interviews, which consisted of seven women and three men, ranging from 30 to 60 years of age. Duration of the interviews varied from 28 min to 1 h 8 min, with a mean duration of 45 min. The results are described in two sections including an inductive thematic analysis and a deductive analysis where the themes are classified into the COM-B framework.

### Thematic analysis

The inductive thematic analysis of transcripts resulted in 10 themes. All participants mentioned the themes *Project management*, *Technological solutions*, and *Efficacy*, while 9 of 10 participants talked about *Organizational support*, *Interaction*, *The bigger picture,* and *Professional development*. The remaining themes of *Comprehension of the project*, *Time* and *Motivation* were discussed by 7–8 participants. Table 3 displays a description of the 10 themes.

**Table 3:** Themes and descriptions

Theme	Description
**Project Management**	Project management includes both how the participants perceived the project management, how they organized the project and the training of the CoP members. The project management was perceived as available, proficient, helpful and accepting which made the participants feel welcome and reassured. Overall the project was perceived as well organized. The training, which included e-learning and learning-by-doing, received positive feedback. Yet, the participants wished for more training and concentrated instruction in the start-up of the CoP.
**Technological solutions**	The need for technological solutions was communicated as both a strength and a weakness of the project. Technological solutions provided flexibility and made live interaction and learning possible. Yet, the majority of the participants expressed frustration with technological problems and the inability to see each other, which inhibited their contribution in project activities.
**Efficacy**	Efficacy describes the informants’ challenges to performing the project. Several reported a lack of previous experience with research methodology, which was linked to a feeling of high level of difficulty. Reading, discussing and receiving training in their second language (English) was also seen as a challenge. Participants’ ability to understand and employ research was perceived as a prerequisite for success.
**Support from participant’s organization**	The majority of the participants discussed how the project interacted with their regular job. A widely held concern was whether their employers allocated sufficient resources to the project and, in particular, time to attend and work on aspects of it. Participants also expressed a need for the employer organization to be supportive of the project and implementation of evidence-based practice for the project to be successful.
**Interaction**	Interaction and collaboration among participants was perceived as an essential part of the project. Several reported that they appreciated learning from each other, discussing and building relationships. However, these functions had been lacking so far. This was mainly due to using teleconferences as the main medium for interacting. Teleconferences were perceived as a challenging format because of: the large group size; not being able to see each other; not having met before; and language barriers. Thus, participants called for an earlier physical meeting, as well as additional future meetings and alternative ways to communicate to facilitate interaction.
**The bigger picture**	This theme describes the participants’ reflections on the project and RKR’s role in relation to the rehabilitation field and the society. The participants expressed confidence in RKR and the importance of the project in improving the rehabilitation field, which some mentioned was too little evidence-based today. For this to happen it was perceived as essential to ensure quality and intelligibility of the research summaries. Additional strengths of the project were to increase knowledge sharing and network building across institutions. Some mentioned that the rehabilitation field previously had received limited funding and this could be a threat to the project.
**Self-development**	The majority of the participants felt that a positive outcome of participating in the project was developing their professional competence, the opportunity to learn from practitioners from other institutions, and increasing confidence in their own practice.
**Comprehension of project**	Comprehension of the project represents the uncertainty that many of the participants felt regarding the overall project and what their role and tasks were. They felt they knew the primary purpose of the project, but experienced uncertainty regarding more specific objectives, timelines, and what was to be expected of them to contribute.
**Time**	Several participants felt the project was time consuming, especially in combination with their regular work responsibilities. Thus, a challenge was to manage and prioritize their own time to the project and make use of that time effectively. Most of the participants regarded the project as a part of their job and were reluctant to spend their leisure time working on the project. Some also reported that they felt it took a long time before they started actively working on the project.
**Motivation**	Several participants mentioned being motivated by participating in a new project that extends beyond their own organization. It was perceived as meaningful and motivating to write research summaries that were relevant to their own practice. Some also mentioned being motivated by enthusiastic fellow participants and the opportunity to work with the competent professionals managing the project.

### Classification of themes in COM-B

After conducting the inductive thematic analysis, each theme was mapped within the COM-B framework to identify important factors that could impact continuing active participation. Two themes were mapped onto **Capability,** two were related to **Motivation,** and four themes were placed within **Opportunity**. Two themes, *Time* and *The bigger picture,* did not fit within a single component in the COM-B framework but mapped to more than one. Table 4 displays the 10 themes and their placement within the COM-B framework.

**Table 4:** Classification of themes in COM-B

COM – B	Themes (*n* = reporting themes)	Quotes
**Capability** *The individual’s psychological and physical capacity to engage in the activity concerned*	Comprehension of project (*n* = 8)	*“What I could wish for more knowledge about before I entered [the project], is more what is expected of you as a knowledge expert, what this year will involve sort of.”* *“[it’s] been a bit difficult to know what the whole thing is about. It’s a bit like you throw yourself in it, and you don’t know really what you’re supposed to do, where to start. […] Also it’s not so easy to allocate time, when one really doesn’t know where to begin.”*
	Efficacy (*n* = 10)	*“I think it has been difficult […] when I took my education, it didn’t have that research bit in it at all.”* *“There is a lot of difficult, such research terms? With like, standard error and measurement and a lot! Lots of things you have to familiarize yourself with, a lot of numbers, and things that, at least for me, takes a little time to remember what everything means.”*
**Opportunity** *Factors not related to individuals, that enables the target behaviour*	Technological solutions (*n* = 10)	*“The technical hasn’t worked ideally […]. It turns out to be somewhat challenging to have lessons streamed from the US. It has its value of course, and it’s been good. But it has some technical challenges.”*
	Organizational support (*n* = 9)	*“[The project] is organized so we can get things done, but it depends on the employer, how much workload there is […] If you get allocated time to [the project] […] So, it’s both up to me, and my employer, if I will be able to contribute as much as the project requires.”* *“It is about [the project] being rooted with leadership. That they give you time to take part in it and think it is important what you are doing, and that they back you up in doing it in a good way.”*
	Interaction (*n* = 9)	*“Face-to-face communication is easier in many ways, rather than through phone or a screen. So, if you start in the other end, instead of having the physical meeting long after project start up, you could rather arrange it in the beginning […] and switch to teleconference after a while. It is easier to talk to people by phone when you know their faces.”* *“It’s exciting to hear about how others work, and what they think. That you can benefit from the experience of others.”*
	Project management (*n* = 10)	*“[the e-leaning courses] requires that you do something in return and that interactivity has been good. There have been these small courses, simple in their nature, but still it requires something in return.”* *“[the project management] have been very helpful and easy to get in touch with. They’ve been very eager to help as soon as something comes up.”*
**Motivation** *Brain processes that energize and direct behavior*	Inspiration (*n* = 8)	*“If [the summaries are] something that you know you’ll benefit from in your daily practice and that will benefit the patient, the motivation gets a lot higher, to sort of really understand and really study thoroughly and really like, yes, dive into it.”* *“Luckily we are two from our institution, so that’s a strength for us as I see it, that we can pull each other a bit. But I would think for someone who’s alone from an institution and participate in this, if you then struggle with staying motivated, then it’s a threat just to be on your own. Such brief inspirational gatherings would then be useful.”* *“It is important that everybody are able to read research on a decent level in order to participate further. […] Both that everyone is able to and also wants to learn it. […] That you have a personal motivation to get up to the right level. That is a prerequisite for us to be able to succeed.”*
	Professional development (*n* = 9)	*“There is a focus on knowledge, so I think it’s informative, that it’s exciting to learn something new […] To learn about ways to evaluate articles for example, or learning to evaluate research, I think is interesting. So self-development in relation to that.”*
**Multi-component themes**	Time (capability and opportunity) (*n* = 8)	*“We are struggling with allocating the time to do [the project], and I think that will be a challenge ahead as well, that we have to sort of prioritize it and make time to do what needs to be done.”* *“At the time I was asked to, it was a lot to do at work then, so there was simply no time or capacity to work with it during work hours. And then you have other things that you’re doing home, so it’s not always time to sit with at home and if so, then it should perhaps have been more organized for if you are going to work with it at home then you have to write hours, since it is a part of the job.”*
	The bigger picture (capability, opportunity, and motivation) (*n* = 9)	*“[…] and together strengthen the whole rehabilitation field. Because it is a field which somehow falls onto the back burner so to speak, regarding being prioritized, focused on in economic terms by politicians and everything. That we manage to raise [the field] so we have something more to turn the table with, so we are heard and get to show the good effects, and why it is important to focus on. That it is socio-economically sensible […] to actually utilize more documented knowledge and be more systematic in the work we are doing. Then it’s perhaps easier to convince those who hold the money bag and the power and stuff like that.”* *“If we don’t do a good job […] then you might as well not do it at all. The whole point is that the research summaries produced are done thoroughly and have a good quality, because otherwise it might be misleading, maybe things go out to the clinics which shouldn’t be there.”*

Note: definitions in italics taken from Michie et al. 2011 [30] (p. 4)

#### Capability

The themes *Comprehension of the project* and *Efficacy* were related to participants’ capability to perform in the project. Both are associated with psychological capabilities, which refer to “knowledge or psychological skills, strength or stamina to engage in the necessary mental processes.” [31] A key ambition for this project was to involve Knowledge Experts in the development of summaries of evidence. After participating in the project for 3 months, several participants felt the project was challenging and expressed a need for more knowledge and individual skills to successfully achieve the CoP goal.

The theme of c*omprehension of the project* was related to a need for more knowledge, as the participants expressed uncertainty regarding their role and tasks, overall timeline and specific objectives in the project.

For example, some participants expressed a need for having expectations clarified:

*“What I could wish for more knowledge about before I entered [the project], is more what is expected of you as a knowledge expert, what this year will involve sort of.”*

This uncertainty made it difficult for some participants to know where to start or how to structure time to the project:

*“[it’s] been a bit difficult to know what the whole thing is about. It’s a bit like you throw yourself in it, and you don’t know really what you’re supposed to do, where to start. [ … ] Also, it’s not so easy to allocate time, when one really doesn’t know where to begin.”*

*Efficacy* was related to knowledge and psychological skills. Over half the participants considered their lack of experience with research methodology and the high level of difficulty to review literature as potential barriers to success. As some participants remarked:

*“I think it has been difficult [ … ] when I took my education, it didn’t have that research bit in it at all.”*

*“There is a lot of difficult, such research terms? With like, standard error and measurement and a lot! Lots of things you have to familiarize yourself with, a lot of numbers, and things that, at least for me, takes a little time to remember what everything means.”*

The use of the English language was a perceived challenge, as many participants felt this may limit comprehension and their ability to contribute to the discussions. However, others saw the practice of English as an opportunity to improve the use of the language.

*“[the language] is both a weakness and a strength, because it demands you to extend your horizon and improve your language qualifications and everything, so I can see it as a strength, but it is a weakness in that it takes more time, at least for me, it demands a bit more work to create an understanding.”*

These challenges regarding efficacy were mentioned by some to affect participants’ motivation as well:

*“It’s demanding to sit and read article upon article, and to I understand what I’m reading [...] having to concentrate the whole time and having to read in English and interpret what it says [ … ] It has a lot to say for the motivation [...] if there’s things you don’t understand and you’re stuck and you can’t get answers then it’s not very motivating to work with it, neither at work or at home.”*

#### Themes related to motivation

Two themes that were related to motivation mapped onto the reflective component of motivation: evaluations, planning, and decision-making. These were *Inspiration* and *Professional development.* The theme *inspiration* involves factors that the participants explicitly mentioned as important as a stimulus to participate in the project. Some participants felt the project’s purpose of writing research summaries was meaningful, while others mentioned the importance of inspiration from other CoP members. For example,

*“If [the summaries are] something that you know you’ll benefit from in your daily practice and that will benefit the patient, the motivation gets a lot higher, to sort of really understand and really study thoroughly and really like, yes, dive into it.”*

Other participants mentioned the importance of inspiration from other group-members. For example, one participant stated:

*“Luckily we are two from our institution, so that’s a strength for us as I see it, that we can pull each other a bit. But I would think for someone who’s alone from an institution and participate in this, if you then struggle with staying motivated, then it’s a threat just to be on your own. Such brief inspirational gatherings would then be useful.”*

Many participants focused on their own motivation to take part in the project, and some believed it could be a threat for the project if other participants were not sufficiently motivated.

“*It is important that everybody is able to read research on a decent level in order to participate further. [ … ] Both that everyone is able to and also wants to learn it. [ … ] That you have a personal motivation to get up to the right level. That is a prerequisite for us to be able to succeed”.*

In regards to *professional development*, 9 of 10 participants felt they would learn and develop as professionals through involvement in the project. This was a major motivator for several of the participants:

*“There is a focus on knowledge, so I think it’s informative, that it’s exciting to learn something new [ … ] To learn about ways to evaluate articles for example, or learning to evaluate research, I think is interesting. So self-development in relation to that.”*

#### Themes related to opportunity

Four themes reported were related to factors outside the individual. The theme *Technological solutions* was related to the physical environment, while the themes *Organizational Support*, *Interaction,* and *Project management* were mapped onto both the physical and social environment.

*Technological solutions* enabled real-time learning and communication, despite the geographic location of CoP participants. Some mentioned the flexibility of technology as an enabler, but others felt they were a problematic barrier.

“*The technology hasn’t worked ideally [ … ]. It turns out to be somewhat challenging to have lessons streamed from the US. It has its value of course, and it's been good. But it has some technical challenges”.*

#### Support from the participants’ organization

A frequent concern was related to the employer organization’s attitude and allocation of resources to the project. Reliance on clinicians’ spare time for CoP participation was cited as a barrier unless time was given within working hours. Thus, having allocated time to work on project-tasks was considered a critical factor for success.

*“[The project] is organized so we can get things done, but it depends on the employer, how much workload there is [ … ] If you get allocated time to [the project] [ … ] So, it's both up to me, and my employer, if I will be able to contribute as much as the project requires”.*

As described by one participant, support and acknowledgment from the organization were also deemed important.

*“It is about [the project] being rooted with leadership. That they give you time to take part in it and think it is important what you are doing, and that they back you up in doing it in a good way”.*

Another theme related to opportunity was *Interaction* within the CoP. Participants expressed concern about the lack of discussions, especially during teleconferences. The participants had an in-person meeting 3 months after project initiation, and many commented that not meeting each other in advance contributed to the lack of interaction.

*“Face-to-face communication is easier in many ways, rather than through phone or a screen. So, if you start in the other end, instead of having the physical meeting long after project start up, you could rather arrange it in the beginning [ … ] and switch to teleconference after a while. It is easier to talk to people by phone when you know their faces.”*

Developing relationships with other CoP members was identified as a strength in regards to expanding social networks, discussing the project tasks and learning from others.

“*It’s exciting to hear about how others work, and what they think. That you can benefit from the experience of others.”*

All participants mentioned aspects related to the *project management*. In general, perceptions of the management were positive, both regarding interaction with the CoP and project planning, organization, and training. Frequently reported positive characteristics of the project management were openness, welcoming and trustworthy.

*“[the project management] have been very helpful and easy to get in touch with. They’ve been very eager to help as soon as something comes up.”*

The e-learning resources were also described as useful.

*“[the e-leaning courses] requires that you do something in return and that interactivity has been good. There have been these small courses, simple in their nature, but still it requires something in return.”*

#### Multi-component themes

*Time* and *The bigger picture* did not readily map onto a single COM-B framework component, but could rather be placed on several. *Time* included the participants’ capability to manage one’s own time, and also external factors such as difficulty completing activities in addition to regular work responsibilities. Thus, this theme can be mapped onto both capability and opportunity.

*“We are struggling with allocating the time to do [the project], and I think that will be a challenge ahead as well, that we have to sort of prioritize it and make time to do what needs to be done.”*

*“At the time I was asked to, it was a lot to do at work then, so there was simply no time or capacity to work with it during work hours. And then you have other things that you’re doing at home, so it’s not always time to sit with it at home and if so, then it should perhaps have been more organized for if you are going to work with it at home then you have to write hours, since it is a part of the job.”*

In regards to *the bigger picture,* which related to all three factors in the COM-B model, several participants reflected on the benefit of project collaboration across institutions as one that would improve capability through the collective work of the group and opportunity through the impact on the health region. As a motivation, some also reflected on the importance of producing summaries of high quality since others in the health region will rely upon them.

## Discussion

This project describes the early development of a CoP focused on knowledge synthesis and adaptation, and factors that may contribute to the success of the CoP. Clinicians reported relatively low evidence-based practice confidence on the EPIC survey, which is similar to other studies on examining evidence-based practice confidence of among physical therapists [22]. Ten themes of factors that may impact CoP success were mapped to the COM-B model. These included project management, technological solutions, efficacy, organizational support, interaction, the bigger picture, self-development, time, and motivation.

*Capability*, as described as the individual’s psychological and physical capacity to engage in the project, included themes of comprehension of the project, efficacy, and time to participate. These results are similar to another PM&R CoP that developed guidelines for the treatment of low back pain [15], and suggest that clearly articulated project goals and CoP member roles may facilitate CoP success. Similarly, McCreesh and colleagues also demonstrated that critical appraisal efficacy and dedicated time to participate may be a barrier to success in the CoP [19]. Mechanisms to provide education and training to ensure CoP members have the skills may also improve and contribute to the success of the group. In studies of physical therapists with similar EPIC scores, an EPIC score increase of 56 to 74.3% was demonstrated after a 6-month active program that included management support and electronic resources, 2-day evidence-based practice workshop, 5 months of synthesizing research into locally actionable practice behaviors, and agreement to implement the best practices [22, 34]. This suggests a program, such as the Knowledge Expert project, may contribute to improved efficacy. However, these data also suggest the CoP needs to ensure its members have dedicated time to participate. This set of themes may not be specific to the CoP, but rather factors that may be important to engage individuals in any project.

Similar to other PM&R studies, the Knowledge Experts identified *opportunities* that included technological solutions, organizational support, time, interaction, and project management. Technological solutions were also identified as a primary barrier to the success of an online forum for peer collaboration, mentoring and networking [17]. Organizational support and interaction were described as potential barriers in a CoP of pediatric occupational and physical therapies in school districts [16]. Enablers included having management support and time provided within the workday to complete CoP tasks, face-to-face meetings, small group size (no more than 10 people), active facilitation, professionalism, and mutual respect for CoP members [16]. Collectively, these results suggest that important factors for CoP success may include a reliable and easy-to-use technology infrastructure, time and incentives to participate in the project provided by the employer of the CoP member, group interaction that includes face-to-face communication, and supportive and interactive project management. While face-to-face communication appears to be important, future studies should examine whether this can be achieved by using online video conferences.

*Motivation*, as identified by the Knowledge Experts, included inspiration and professional development. The CoP members found inspiration to participate in the project through the benefits to clinical practice and patients, and their colleagues. They also felt, because of this inspiration, it was necessary to produce high-quality work. Also perceived as a motivator in other CoPs [16–19], clearly defining the potential impact of the CoP on each of these levels may also motivate its members.

The use of the English language in the readings and discussions was a perceived challenge as well as an opportunity since English was a second language for all participants. In a recent systematic review of barriers to evidence-based practice across primary health care, secondary and specialist care (hospitals), rehabilitation care, and medical education, language barriers were among the most commonly cited across the studies [35]. Research in physician practice has identified that reading articles in a non-native language may result in lower comprehension and may also require increased time to read the article [36]. Other studies have indicated that clinicians often prefer to read articles published in journals of their native language [37]. While none of these articles specifically evaluated barriers for native Norwegian speakers, data from this project indicate that language may also be a barrier in this context. More research on this possible barrier needs to be conducted, especially since the majority of peer-reviewed articles are published in English [37]. In addition, research on ways to overcome this barrier for non-native English speakers should be conducted to facilitate use of evidence in practice.

When discussing potential threats to CoP success during the interviews, seven individuals reported a threat of time and 9 indicated a threat of organizational support. Other PM&R studies have identified the need for strong organizational support [15, 16, 20] and time for CoP related meetings and work [16, 17, 19]. In a study by Tilson and colleagues, the COM-B model was used to describe reasons for lack of translation of best practices into routine behaviors. While capacity and motivation were addressed by improving evidence-based practice knowledge and skills and creating an energized culture aimed to improve patient care, the opportunity may have been missed because organizational support for implementation and sustainability was not garnered [34]. These data collectively suggest that organizational support of CoPs and allocating time for participation may be critical factors for the success of maintaining CoP participation and application of the knowledge gained. In addition, these data suggest the project management team should also regularly communicate with organizational leaders about project goals, expectations, and progress with organizational leaders.

The CoP members cited professional development, impact on patients, and the potential for impact on others in the health region as the primary reasons for continuing in the group. Also cited as the strongest motivator to participate in a CoP that focused on evidence-based practice recommendations for physical therapists [19], these data suggest the importance of ensuring that members understand the potential impact of the CoP on themselves, patients, and the healthcare community.

### Limitations

There are many limitations to this study. CoP members were recruited from the southeastern region of Norway, and clinicians volunteered to participate in the CoP. Therefore, this sample may represent a group of clinicians who were particularly interested or motivated to achieve the CoP aims. We used a convenience sampling strategy in this project, which may limit the generalizability of these findings to other CoPs. Further, the data presented here reflect the CoP members’ perceptions, which may or may not represent CoP members’ actions or behaviors. They also represent perceptions of the Knowledge Experts as the CoP was developed, however, they do not include information about how these perceptions changed over time. The information provided by the Knowledge Experts informed the development of the CoP, however, this study does not provide the outcomes of the CoP activities. More research is needed to determine whether the recommendations provided result in successful CoPs.

## Conclusions

This study describes the experiences of the early phase of development of a CoP focused on knowledge synthesis and adaptation in the field of PM&R. The project consisted of a CoP that participated in online education and training on critical appraisal and knowledge translation, bi-weekly online meetings, and development of a summary of evidence. Importantly, interview results suggest a few critical components of establishing a CoP in healthcare include addressing capability, opportunity, and motivation. In summary, clearly articulated project goals and participant expectations are necessary, in addition to education and training to ensure the CoP participants have the necessary knowledge and skills to be successful. Reliable and easy to use technology solutions, organizational support of the CoP member and the project, interaction that includes face-to-face communication, and good project management should be present. The potential impact of the CoP on the member, patients, and others should be clear to inspire participation. Lastly, CoP members should have dedicated time and organizational support to participate in related activities.

## Supplementary Information

**Additional file 1.** Interview Guide in Norwegian and English.

## Data Availability

The datasets generated and/or analysed during the current study are not publicly available due to the small sample size and the possibility of identifying participants’ responses. The members of the CoP are known to the public; therefore, we must maintain the confidentiality of their answers.

## Abbreviations

COM-B: Capability, Opportunity, and Motivation Model of Behaviour
CoP: Community of Practice
EPIC: Evidence-Based Practice Confidence scale
PM&R: Physical Medicine and Rehabilitation
