# Supplementary material for: A qualitative study exploring contributors to the success of a community of practice in rehabilitation
Source: BMC Med Educ. 2021 May 17;21:282. doi: 10.1186/s12909-021-02711-x (PMC8130156; doi:10.1186/s12909-021-02711-x)
Supplement: Supplementary file 1 — Additional file 1. Interview Guide in Norwegian and English. [file 12909_2021_2711_MOESM1_ESM.pdf]

A Qualitative Study Exploring Contributors to the Success of a Community of Practice in Rehabilitation

Jennifer Lynn Moore, Cato Bjørkli, Richard Tidemann Havdahl, Linn Lien Lømo, Mari Midthaug, Marita Skjuve, Jan Egil Nordvik

Supplementary file 1. Interview Guide in Norwegian and English

Norwegian Interview Guide

Hilse og skape relasjon.

- Hilse med navn, og introdusere seg selv med navn.
- Smil og blikk-kontakt
- Still uformelle åpne spørsmål, vis interesse for svar, gjerne oppfølgingsspørsmål
- Fortell noe uformelt selv, gjerne basert på noe informant har sagt. Gi gode beskrivelser, vær en god rollemodell for hvordan man er informant.
- Øv allerede på ikke å avbryte, ha rolig stemme og tempo, behagelig atmosfære, interesse for informanten, ikke heng deg opp i formalia eller papirer.
- Fortsett med denne fasen HELT til informanten slapper av.

Motiver til å gå inn i informantrollen.

- ”Har du noen tanker om hva vi vil prate om idag?”
- Forklar formålet med dette intervjuet – forklar hva vi mener med KE-prosjektet
- Forklar at intervjuet ofte har noen antakelser med seg – at intervjueren skal styre samtalen og ha konkrete spørsmål, informanten skal kun svare på spørsmålet og ikke gå utenfor dette. At informanten skal være presis og sikker i sin sak, ikke plapre i vei eller være vag eller ombestemme seg midt i en setning. I samtaler generelt er det ansett som ”høflig” å ikke gå i detaljer eller prate i vei om noe som kanskje virker uinteressant for den andre parten. Alt dette er ganske motsatt i vår type intervju!
- I intervju lar man ofte være å fortelle noe fordi man tenker at dette gjelder bare akkurat meg og min spesifikke situasjon, eller dette gjelder bare akkurat min gruppe og har ikke så mye å si i prosjektet som helhet, men vi ønsker at du forteller så mye som mulig av dine opplevelser i prosjektet. Ditt unike synspunkt er det vi er ute etter.
- Forklar rollefordelingen – informanten skal styre intervjuet og prate mest mulig, ta seg god tid om de trenger å tenke. Det kan godt tenkes at informanten gjør refleksjoner her og nå som hun/han ikke har tenkt grundig gjennom tidligere. Det er helt greit – man behøver ikke være helt sikker i alt man opplever og tenker.
- Forklar at informantrollen er vanskelig og krever motivasjon og konsentrasjon til å ville dele så mye som mulig og gjøre refleksjoner.
- Spør om informanten har noen spørsmål. Deretter spør om hun/han er klar til å begynne.

Om formålet med intervjuet.

Målet er å få kjennskap til hvilke faktorer som oppleves av betydning for KEs i dette prosjektet. Vi vil hjelpe RKR å finne ut hva de bør gjøre får å få dette prosjektet til å bli en suksess. Hva de har gjort bra og bør fortsette med. Hva de har gjort mindre bra og bør endre på. Hvilke muligheter de burde vurdere som kan gjøre prosjektet enda bedre. Hvilke mulige trusler de må være obs på for å unngå at prosjektet møter problemer. Vi mener dere som deltar i prosjektet er hovedpersonene og de som kjenner best på hva som fungerer og ikke fungerer. Derfor ønsker vi å få høre om deres opplevelser og vurderinger i prosjektet. Dere er 10 stykker som deltar så hver enkelt av dere sitter med veldig viktig informasjon for RKR. Dine opplevelser og tanker og vurderinger kan ha stor betydning for hvordan prosjektet utvikler seg videre.

Intervju.

- Kan du fortelle om prosjektet du er med i?
- Hva tenker du er din rolle i prosjektet?

I første omgang vil vi høre om hvordan du har opplevd prosjektet frem til nå. Prøv å tenke på tiden helt fra du først ble involvert og frem til i dag.

- Kan du fortelle om hva som har vært bra i KE-prosjektet til nå? Vi kan kalle dette styrkene ved KE-prosjektet.
    - Du nevnte … Fortell hvorfor du mener dette er viktig eller evt ikke så viktig.
    - Du nevnte … kan du fortelle mer om dette?
    - Du nevnte … kan du gi noen konkrete eksempler som illustrerer dette?
- Kan du fortelle meg om hva som ikke har vært så bra i KE-prosjektet til nå? Vi kan kalle dette svakhetene ved KE-prosjektet.
    - Du nevnte … Fortell hvorfor du mener dette er viktig eller evt ikke så viktig.
    - Du nevnte … kan du fortelle mer om dette?
    - Du nevnte … kan du gi noen konkrete eksempler som illustrerer dette?

Nå vil vi at du tenker fremover i tid, videre i prosjektet.

- Kan du fortell meg om det du ser som muligheter for å forbedre KE-prosjektet? Vi kan kalle dette mulighetene for bedring av KE-prosjektet.
    - Du nevnte … Fortell hvorfor du mener dette er viktig eller evt ikke så viktig.
    - Du nevnte … kan du fortelle mer om dette?
    - Du nevnte … kan du gi noen konkrete eksempler som illustrerer dette?
- Kan du fortelle meg om hva du anser som hindringer for at KE-prosjektet skal bli vellykket? Vi kan kalle dette truslene i KE-prosjektet.
    - Du nevnte … Fortell hvorfor du mener dette er viktig eller evt ikke så viktig.
    - Du nevnte … kan du fortelle mer om dette?
    - Du nevnte … kan du gi noen konkrete eksempler som illustrerer dette?

English Interview Guide

Greet and create a relationship.

- Greet by name and introduce yourself by name.
- Smile and make eye contact.
- Ask informal open-ended questions, show interest in answers, preferably follow-up questions.
- Tell something informal yourself, preferably based on something the informant has said. Give good descriptions, be a good role model for how to be an informant.
- Do not to interrupt, have a calm voice and tempo, pleasant atmosphere, interest in the informant, do not get hung up on formalities or papers.
- Continue with this phase UNTIL the informant relaxes.

Provide an explanation of the informant role.

- "Do you have any questions about the discussion we will have today?"
- Explain the purpose of this interview, describe the definition of the term “KE project”
- Explain that interviewer should direct the conversation and asks specific questions. The informant should only answer the question and not go beyond this. The informant should be precise and confident in the responses.
- Explain that we want to learn about the informant’s experiences in the project, and that the informant a unique point of view is important.
- Explain the division of roles. The informant should talk as much as needed and take the time that is needed to think about responses.
- Explain that the informant role is difficult and requires motivation and concentration to make reflections and share as much as possible.
- Ask if the informant has any questions. Then ask if he / she is ready to start.

The purpose of the interview.

During this interview, we aim to gain knowledge of that factors that are important for Knowledge Experts in this project. We want to identify ways RKR can make this project a success, what RKR has done well and should continue with, components of the project that should change, opportunities to improve the project, and possible threats to avoid.

We believe the informants, as participants of the project, know what is best for the project and what does not work well in the project. Therefore, we want to hear about the informants’ experiences and perspectives about the project. They can have a great impact on how the project develops further.

Interview.

- Can you tell us about the project you are part of?
- What do you think is your role in the project?

We would like to hear about how you have experienced the project so far. Please think of the time from the time you first got involved until today.

- Can you tell me about what has been good in the KE project so far? We can call this the strengths of the KE project.
    - You mentioned… Explain why you think this is important or possibly not so important.
    - You mentioned… can you tell more about this?
    - You mentioned… can you give some concrete examples that illustrate this?
- Can you tell me about what has not been so good in the KE project so far? We can call this the weaknesses of the KE project.
    - You mentioned… Explain why you think this is important or possibly not so important.
    - You mentioned… can you tell more about this?
    - You mentioned… can you give some concrete examples that illustrate this?

Now we want you to think about the future of the project.

- Can you tell me about what you see as opportunities to improve the KE project? We can call this the opportunities for improvement of the KE project.
    - You mentioned… Explain why you think this is important or possibly not so important.
    - You mentioned… can you tell more about this?
    - You mentioned… can you give some concrete examples that illustrate this?
- Can you tell me about what you consider to be obstacles to the KE project's success? We can call this the threats in the KE project.
    - You mentioned… Explain why you think this is important or possibly not so important.
    - You mentioned… can you tell more about this?
    - You mentioned… can you give some concrete examples that illustrate this?
